# Glycyrrhiza polysaccharide attenuates *Neospora caninum*-induced intestinal epithelial cell damage by the C/EBPβ/IL-17/TNF signaling pathway

**DOI:** 10.3389/fvets.2025.1753653

**Published:** 2026-01-26

**Authors:** Shuai Wang, Sudan Meng, Yongsheng An, Weifeng Qian, Yanbo Ma, Shuai Guo, Cai Zhang

**Affiliations:** College of Animal Science and Technology, Henan University of Science and Technology, Luoyang, China

**Keywords:** C/EBPβ/IL-17/TNF signaling pathway, Glycyrrhiza polysaccharide, inflammatory regulation, intestinal damage, intestinal health

## Abstract

Intestinal epithelial cell (IEC) damage is a crucial event in pathogen-induced intestinal inflammation and systemic pathological responses, and their functional integrity directly affects animal health. This study used bovine intestinal epithelial cells (BIECs-21) and mouse models to examine the protective effects of Glycyrrhiza polysaccharide (GCP) against *Neospora caninum* (*NC*)-induced IEC damage and investigate its underlying mechanisms. *In vitro*, BIECs-21 were infected with *NC* to establish an intestinal epithelial injury model. *In vitro* experiments revealed that GCP pretreatment effectively inhibited *NC* infection-induced decreases in cell viability and lactate dehydrogenase (LDH) release, preserving intestinal epithelial homeostasis. Transcriptomic analysis results showed that *NC* infection activated the interleukin (IL)-17 and tumor necrosis factor (TNF) signaling pathways, increasing the expression of chemokines (CXCL1/2/3) and inflammatory genes (FOSB). In contrast, GCP inhibited the expression of transcription factors CCAAT/enhancer-binding protein β (C/EBPβ) and FOS, reduced pro-inflammatory factors (e.g., IL-6, IL1RAP), and mitigated excessive inflammatory responses. *In vivo* experiments confirmed that low-dose GCP intervention significantly reduced intestinal hemorrhage and edema, decreased parasite loads in intestinal and cerebral tissues of infected mice, and suppressed protein expression of IL-17RA, TNF-*α*, p-C/EBPβ and p-NF-κB in intestinal tissues. These findings demonstrate that GCP mitigates *NC*-induced IEC injury by modulating intestinal immune homeostasis through the C/EBPβ/IL-17/TNF signaling pathway, thus establishing a theoretical basis for developing natural therapeutics against pathogen-induced gut damage.

## 1 Introduction

The intestine is not only a vital organ for nutrient digestion and absorption but also the largest immune organ in the body, performing essential roles in immune barrier maintenance and systemic physiological regulation (1, 2). Thus, ensuring animal intestinal health is one of the key links in safeguarding the healthy development of the livestock industry. Intestinal epithelial cell (IEC), the primary barrier against exogenous pathogens, maintain intestinal homeostasis through tight junction complexes, mucus layers, and antimicrobial peptide secretion systems (3). However, the intestine is susceptible to invasion by pathogens including bacteria, viruses, and parasites, resulting in compromised barrier integrity and systemic immune dysregulation (4).

*Neospora caninum* (*NC*) infection begins with IEC invasion and intracellular proliferation, essential steps for systemic dissemination. However, the endogenous defense mechanisms of IECs against *NC* remain inadequately characterized. Improving intestinal health is a crucial strategy to inhibit intracellular pathogen proliferation and prevent infection progression (5–7). Thus, understanding IEC defense mechanisms and identifying natural compounds that enhance resistance to intracellular pathogens are important for improving livestock productivity and public health security. Although human infections remain undocumented, anti-*NC* antibodies have been detected in humans (8, 9), and transplacental transmission has been demonstrated in primate models (*Macaca mulatta*) (10, 11), indicating potential zoonotic risks.

Glycyrrhiza polysaccharide (GCP), a bioactive compound extracted from the traditional Chinese herb licorice, demonstrates immunomodulatory, antioxidant, anti-inflammatory, and gut microbiota-regulating properties (12, 13). It decreases intestinal permeability and serum levels of pro-inflammatory cytokines (IL-1, IL-6, TNF-*α*) while increasing anti-inflammatory IL-10, thus ameliorating murine colitis (14). Furthermore, GCP modulates gut microbiota composition by enhancing beneficial bacterial growth and inhibiting pathogenic species (15, 16). However, the role of GCP in regulating IEC-intrinsic defense mechanisms against *NC* infection remains unexplored.

This study used BIECs-21 to assess *NC*-induced cellular damage and the protective effects of GCP. Transcriptomic profiling was used to elucidate underlying mechanisms, with *in vivo* experiments validating the findings.

## 2 Materials and methods

### 2.1 Cell culture and treatment

BIECs-21, previously immortalized by our laboratory (17), and Vero cells (African green monkey kidney epithelium, kindly provided by Prof. Lei He, Henan University of Science and Technology) were utilized. *NC* tachyzoites were obtained from Prof. Qun Liu at China Agricultural University.

BIECs-21 were maintained in DMEM (Gibco, USA) supplemented with 10% FBS (Cegrogen, Germany) and 500 μg/mL G418 (Beyotime, China) at 37 °C under 5% CO₂. Vero cells were cultured in DMEM with 10% FBS for *NC* propagation. Infection models were established by inoculating BIECs-21 with *NC* tachyzoites at a 3:1 parasite-to-host cell ratio. For pretreatment experiments, BIECs-21 were incubated with the optimal dose of GCP (1,000 μg/mL, Supplementary Figure 1) for 12 h prior to *NC* exposure.

### 2.2 Cell viability assay

BIECs-21 were seeded in 96-well plates and divided into four groups: control (C), GCP-treated (GCP), *NC*-infected (NC), GCP-pretreated + *NC*-infected (GNC). After 12 h GCP incubation and 4 h *NC* infection (MOI = 3:1), cell viability was assessed using CCK-8 (Solarbio, China). Following reagent addition (10 μL CCK-8 + 90 μL DMEM), plates were incubated at 37 °C for 1.5 h. The absorbance of the supernatant was measured at 450 nm using a microplate reader (Thermo, USA).

### 2.3 Lactate dehydrogenase (LDH) release

BIECs-21 cells were seeded in a 96-well plate and the lactate dehydrogenase (LDH) activity in the culture supernatant was quantified using a Lactate Dehydrogenase Assay Kit (Nanjing Jiancheng, China). After incubation at 37 °C for 1 h, the absorbance of the supernatant was measured at 490 nm to assess membrane integrity.

### 2.4 Transcriptomic profiling

Total RNA from four experimental groups (C, GCP, NC, GNC) was extracted with TRIzol (Ambion, USA). RNA libraries were prepared using NEBNext Ultra II reagents (New England Biolabs) and sequenced on Illumina NovaSeq 6,000 (150-bp paired-end) by Personalbio (Shanghai, China). Differentially expressed genes (DEGs) were identified with |log₂FC| > 1 and *p* < 0.05. Functional enrichment analyses were performed using topGO (v2.40.0) for Gene Ontology and ClusterProfiler (v3.16.1) for KEGG pathways.

### 2.5 Quantitative PCR

Total RNA was extracted from cells or tissues using TRIzol (Ambion). Specific primers were designed and synthesized by Sangon Biotech (Shanghai, China), and the primer sequences can be found in the Supplementary Table 1. cDNA was synthesized using a reverse transcription kit (Vazyme, China). SYBR Green-based qPCR (Vazyme, China) was conducted on a Bio-Rad system(Bio-Rad, USA) with β-actin as endogenous control. Relative expression was calculated via 2^^(-ΔΔCt)^ method.

### 2.6 Animal experimentation

Fifty female Kunming mice (6 ~ 8 weeks old) were housed under controlled conditions (20 ~ 24 °C, 40 ~ 70% humidity, 12 h light/dark cycle) with ad libitum access to food and water. Mice were randomized into five groups (n = 10/group): control (no treatment), *NC*-infected (NC), low-dose GCP (100 mg/kg) + *NC* infected (NC + L), medium-dose GCP (200 mg/kg) + *NC* infected (NC + M), High-dose GCP (400 mg/kg) + *NC* infected (NC + H). GCP was administered via drinking water for 25 days pre-infection. All groups except controls were intraperitoneally inoculated with 1 × 10⁶ tachyzoites/mouse. GCP supplementation continued for 8 days post-infection.

### 2.7 Parasite load quantification

Brain and duodenal tissues collected 8 days post-infection were homogenized for genomic DNA extraction. Absolute qPCR was performed using standardized DNA (200 ng/μL) to quantify parasite load. Primer sequences for *NC*: F: 5’-ACTGGAGGCACGCTGAACAC-3′, R: 5’-AACAATGCTTCGCAAGAGGAA-3′.

### 2.8 Western blot assays

Total proteins extracted with RIPA buffer (Solarbio, China) were separated by SDS-PAGE and transferred to PVDF membranes. After blocking with 5% BSA, membranes were probed with primary antibodies followed by HRP-conjugated secondary antibodies. Signals were detected using ECL substrate (Millipore, USA) and analyzed with Image J.

### 2.9 Statistical analysis

Data are presented as mean ± SEM. Group comparisons employed Student’s t-test (pairwise) or one-way ANOVA with Duncan’s *post hoc* test (SPSS v19.0). Graphical outputs were generated using GraphPad Prism 8. Significance thresholds: *p < 0.05, **p < 0.01, ***p < 0.001.

## 3 Results

### 3.1 Damage to BIECs-21 by *NC* and protective effects of GCP

**Figure 1:**
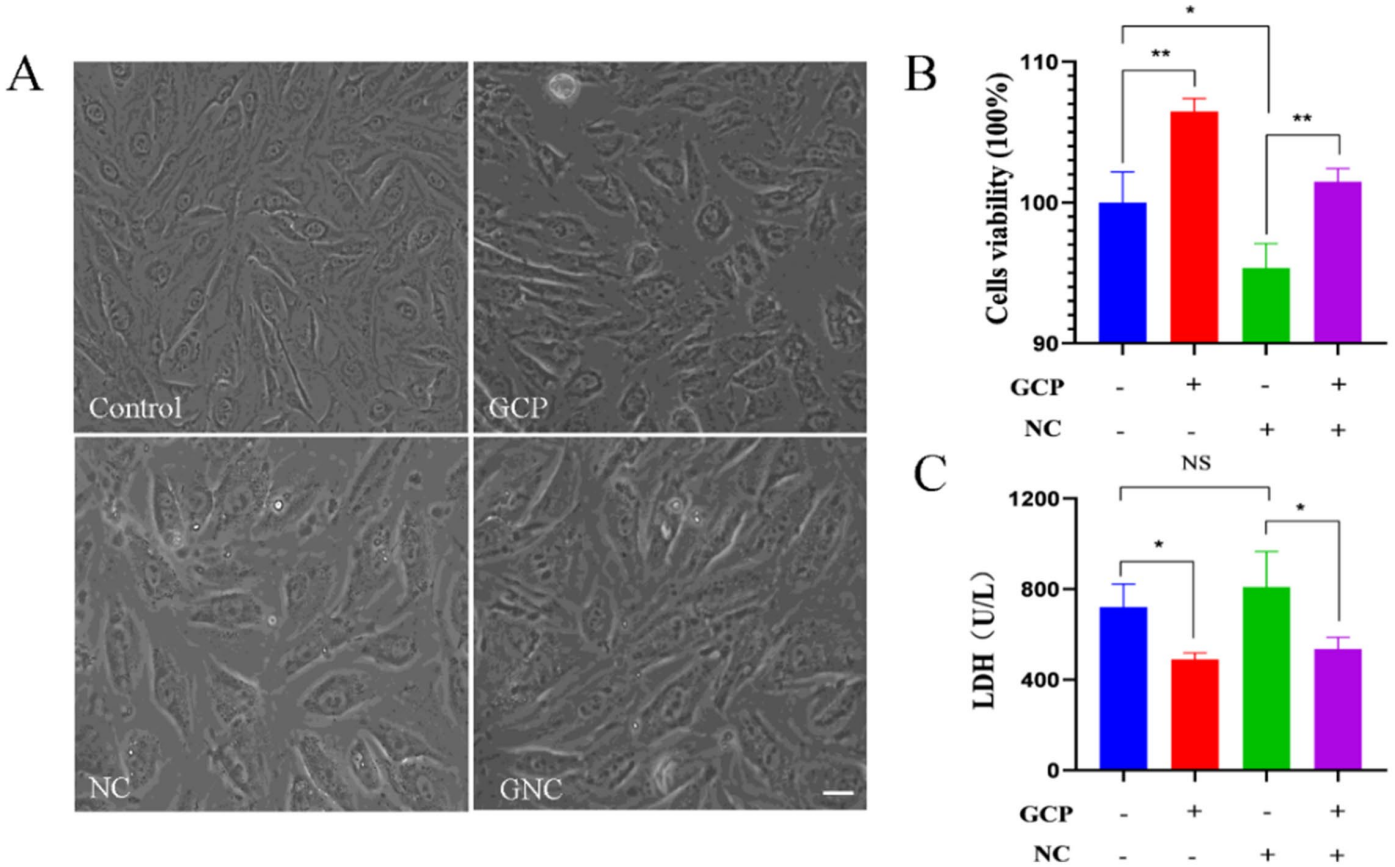
Damage to BIECs-21 by *NC* and protective effects of GCP. **(A)** Morphology of BIECs-21 cells in different treatment groups. **(B,C)** Cells viability and LDH activity of BIECs-21 with *NC* infected for 4 h and pretreated with GCP for 12 h. *p* < 0.01, ***p < 0.001. NS, no significant differences.

No morphological changes were observed in BIECs-21 among control, NC group, GCP group or GNC group (Figure 1A). However, cell viability significantly increased in the GCP group and decreased in the NC group compared to controls. GCP pretreatment markedly inhibited *NC*-induced viability reduction (Figure 1B). Furthermore, LDH activity was significantly lower in the GCP group than in controls, while the NC group exhibited a trend toward elevated LDH. Notably, GCP pretreatment (GNC group) substantially reduced LDH activity relative to the NC group (Figure 1C).

### 3.2 Transcriptomic profiling of differentially expressed genes (DEGs)

**Figure 2:**
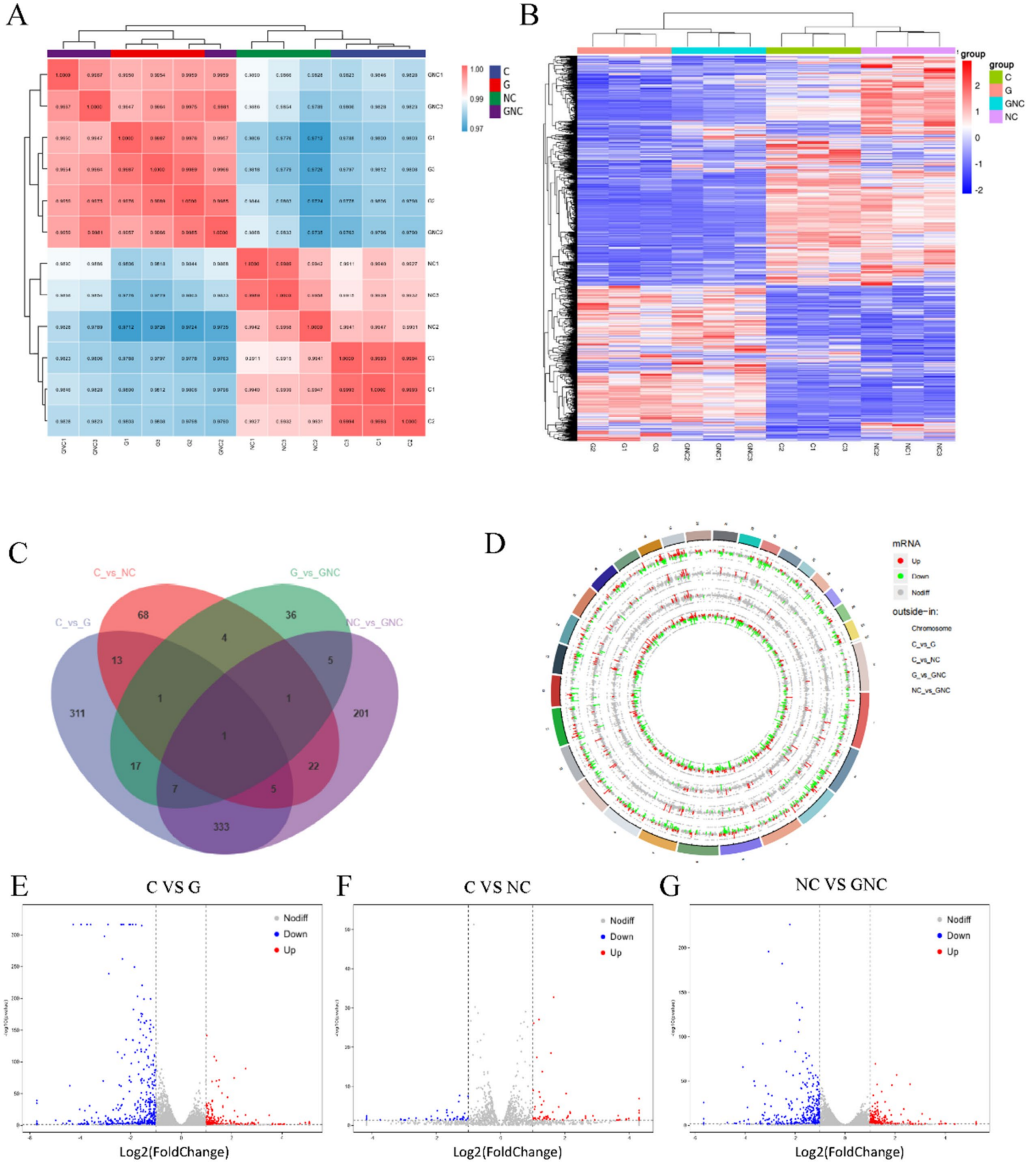
Transcriptome analysis of BIECs-21 pretreated with GCP and infected with NC. **(A)** Correlation analysis of patterns of gene expression in each group. **(B)** A heatmap of DEGs in each group. **(C)** Venn diagram of the number of DEGs in each group. **(D)** Circular visualization of the genomic alterations in BIECs-21 exposed to *NC* and pretreated with GCP. **(E)** A volcanic map of DEGs in control and GCP group. **(F)** A volcanic map of DEGs in control and NC group; **(G)** A volcanic map of DEGs in NC group and GCP-pretreated group.

Transcriptomic analysis revealed high reproducibility and intergroup correlation (Figure 2A). Comparative DEG analysis identified significant differences between G (GCP-treated) vs. C (control), NC vs. C, and GNC vs. NC groups, with pronounced changes in G vs. C and GNC vs. NC (Figure 2B). Specifically, 688 DEGs (226 upregulated, 462 downregulated) were detected in G vs. C, 115 DEGs (69 upregulated, 46 downregulated) in NC vs. C, and 575 DEGs (216 upregulated, 359 downregulated) in GNC vs. NC (Figures 2C–G).

### 3.3 Functional annotation of DEGs

**Figure 3:**
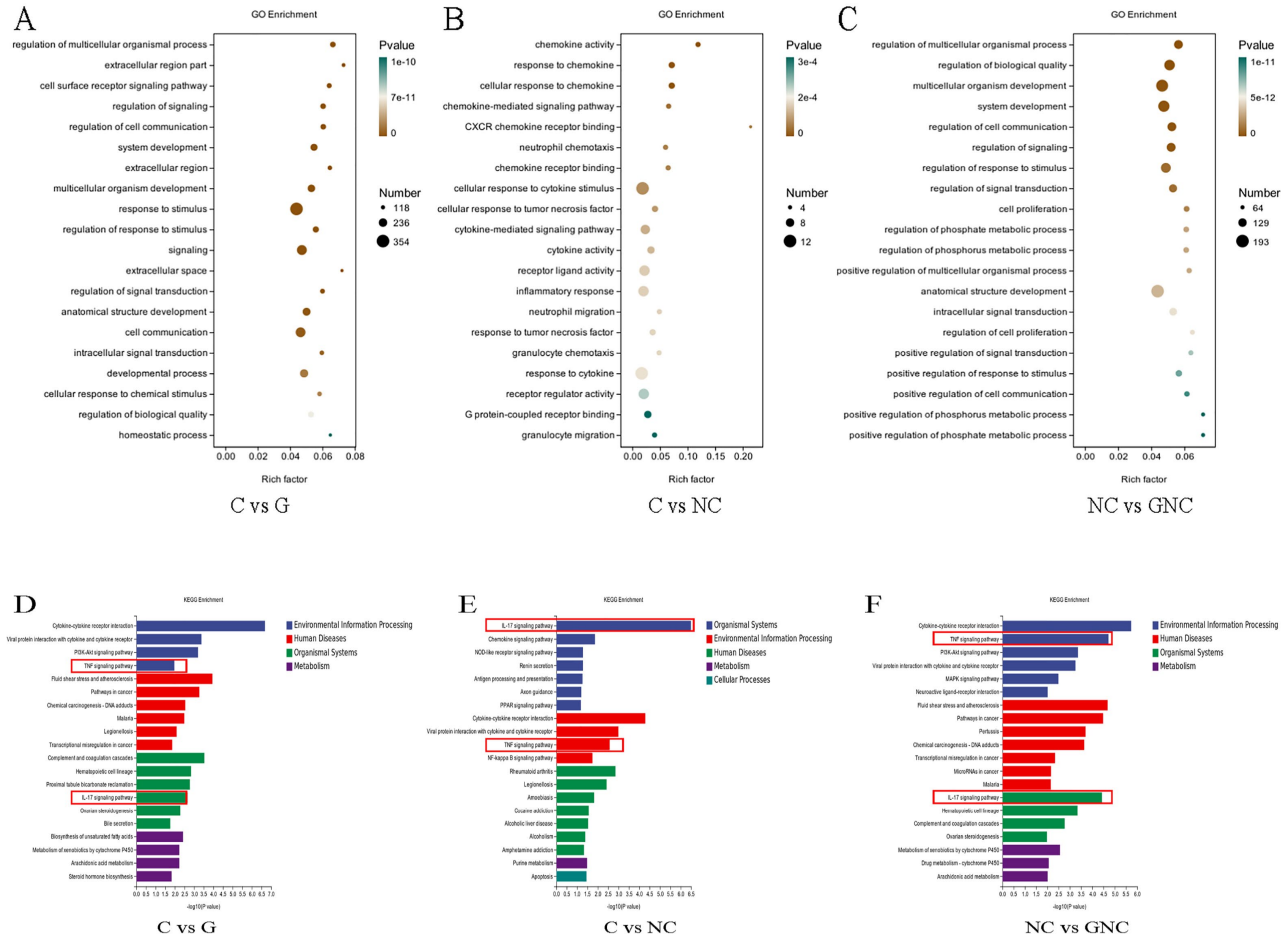
Functional annotation of DEGs. **(A–C)** GO enrichment analysis of DEGs. **(D–F)** KEGG enrichment pathway analysis of DEGs.

GO enrichment analysis indicated that *NC* altered immune-related processes in BIECs-21, including chemokine-mediated signaling, neutrophil chemotaxis and inflammatory response. GCP exerted protective effects by modulating stimulus response regulation, signal transduction and cell proliferation (Figures 3A–C). KEGG pathway analysis highlighted significant enrichment of DEGs in IL-17 and TNF signaling pathways across groups (Figures 3D–F). Cross-comparison of these pathways revealed upregulated inflammatory genes (FOSB) in NC vs. C and downregulated CCAAT/enhancer-binding protein β(C/EBPβ) and FOS in GNC vs. NC.

### 3.4 Validation of DEGs

**Figure 4:**
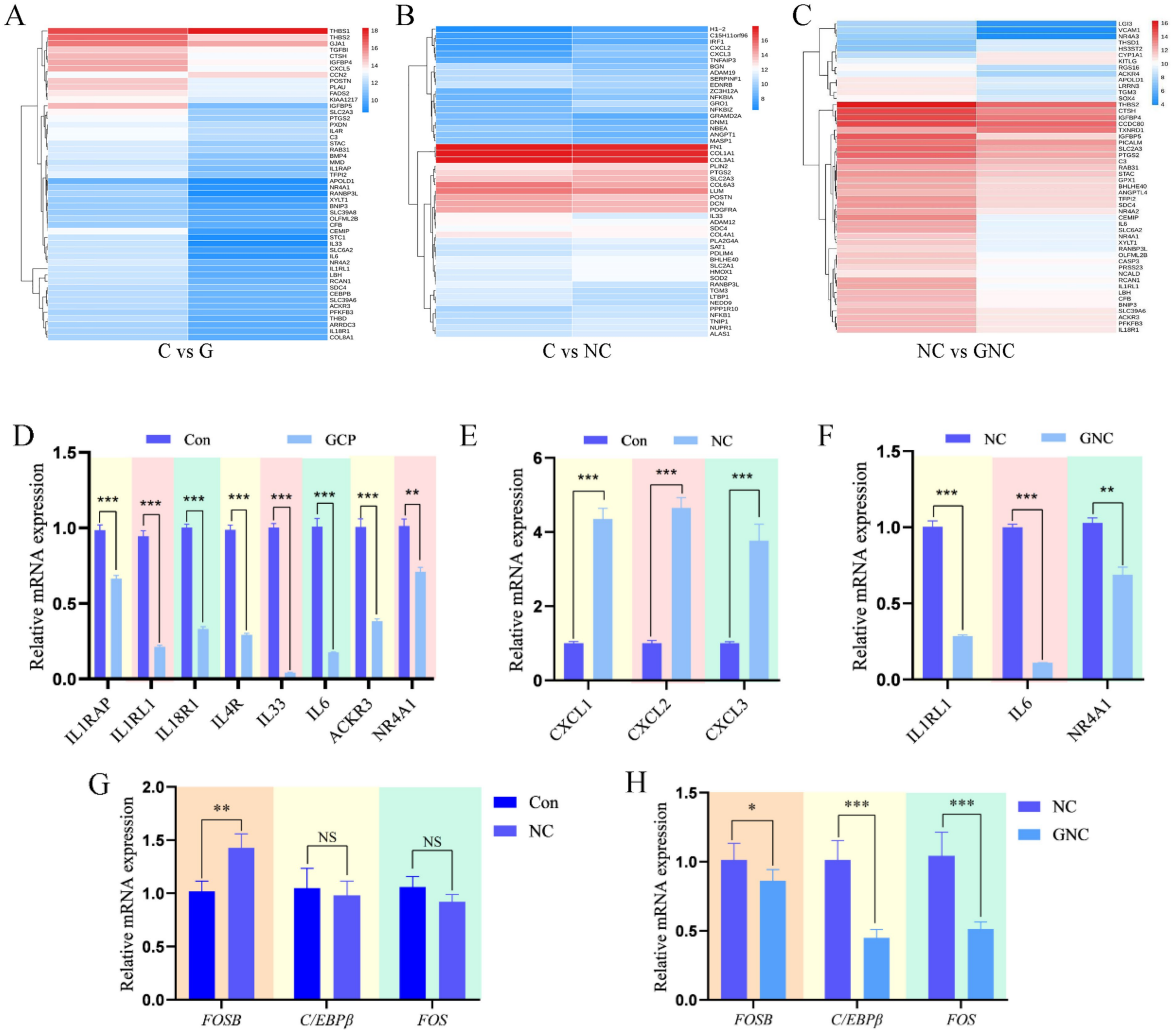
qRT-PCR validation of transcriptome sequencing data. **(A–C)** DEG heatmaps of top 50 in each group. **(D–F)** qRT-PCR validation of differentially expressed genes among groups. **(G,H)** qRT-PCR was used to verify the signaling pathways identified by transcriptome sequencing. The data are expressed in the form of “Mean±SEM.” Statistical significance was calculated by Student’s *t* test. Significance: *p < 0.05, ** *p* < 0.01, *p* < 0.001. NS, no significant differences.

Cluster heatmaps of top 50 DEGs (Figures 4A–C) and qRT-PCR validation confirmed transcriptomic data consistency. Compared to controls, GCP downregulated IL1RAP, IL1RL1, IL18R1, IL4R, IL33, IL6, ACKR3, and NR4A1 mRNA (Figure 4D), and NC upregulated CXCL1, CXCL2, and CXCL3 mRNA (Figure 4E). GNC downregulated IL1RL1, IL6, and NR4A1 mRNA versus NC (Figure 4F). Additionally, FOSB mRNA was elevated in NC vs. C, while C/EBPβ and FOS mRNA were reduced in GNC vs. NC (Figures 4G,H). These results suggest that *NC* exacerbates inflammation via FOSB upregulation, whereas GCP attenuates damage by suppressing C/EBPβ and FOS expression.

### 3.5 GCP alleviates *NC*-induced intestinal damage in mice

**Figure 5:**
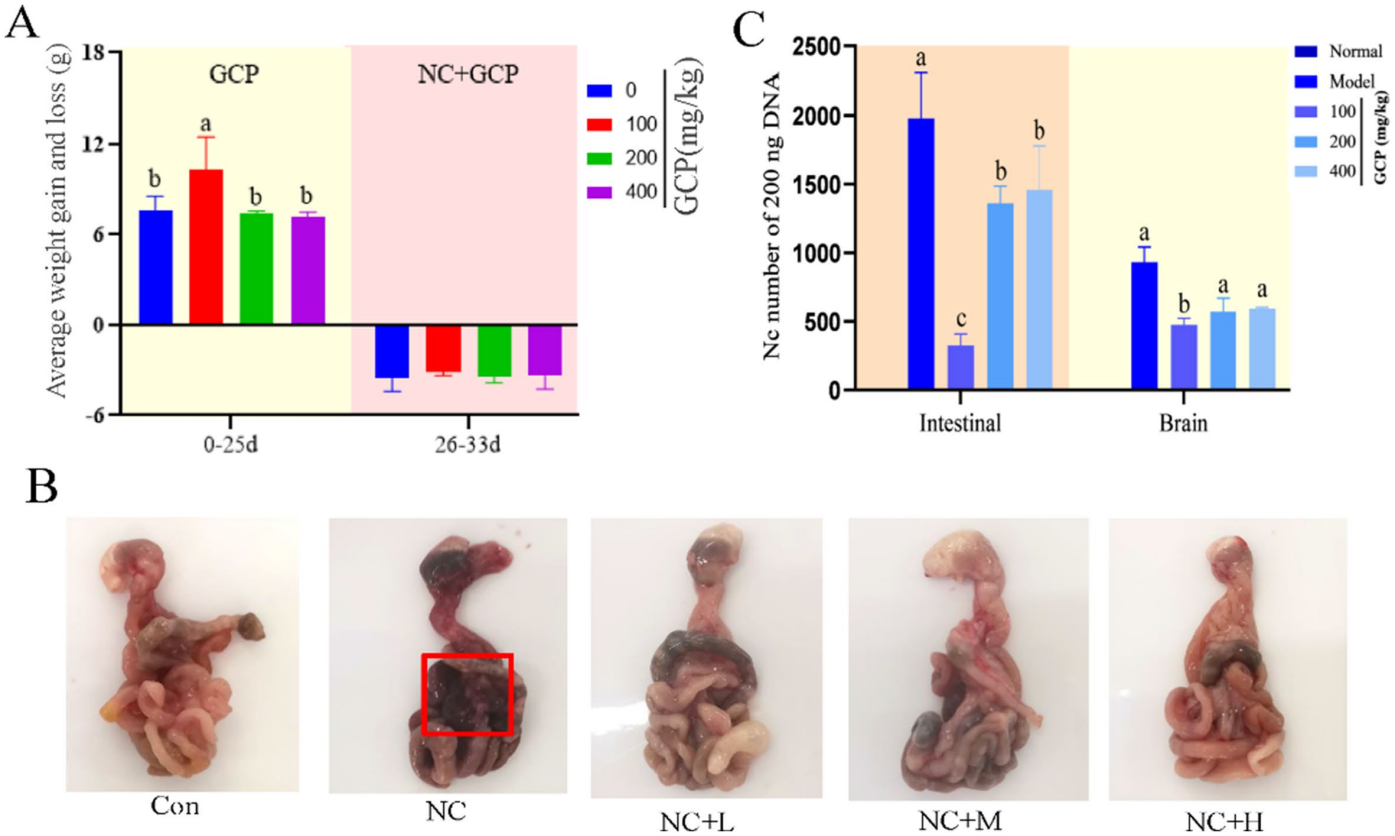
The effect of GCP on body weight and intestinal injury in mice after NC infection. **(A)** Weight gain and loss of mice in each group. **(B)** Intestinal morphology of mice in each group (mesenteric bleeding is marked with a red box). **(C)** Parasites loaded in the duodenum and brain tissues of mice in each group. Statistical significance was calculated by one-way ANOVA with a Duncan test. The same letter in the histogram indicates that there is no significant difference between groups (*p* > 0.05), but different letters indicate significant difference (*p* < 0.05).

To validate the *in vivo* efficacy of GCP, this study established a *NC*-infected mouse model. *In vivo*, low-dose GCP (50 mg/kg) significantly increased body weight gain pre-infection and reduced post-infection weight loss (8 days post-infection) compared to untreated controls (Figure 5A). GCP improved survival rates (Supplementary Figure 2) and mitigated intestinal hemorrhage and swelling (Figure 5B). Further analysis of parasite load in the duodenum and brain tissues of mice across groups revealed that, compared with the NC group, the low-dose GCP treatment significantly reduced parasite load in these two tissues (Figure 5C).

### 3.6 Mechanistic insights into GCP-mediated protection

**Figure 6:**
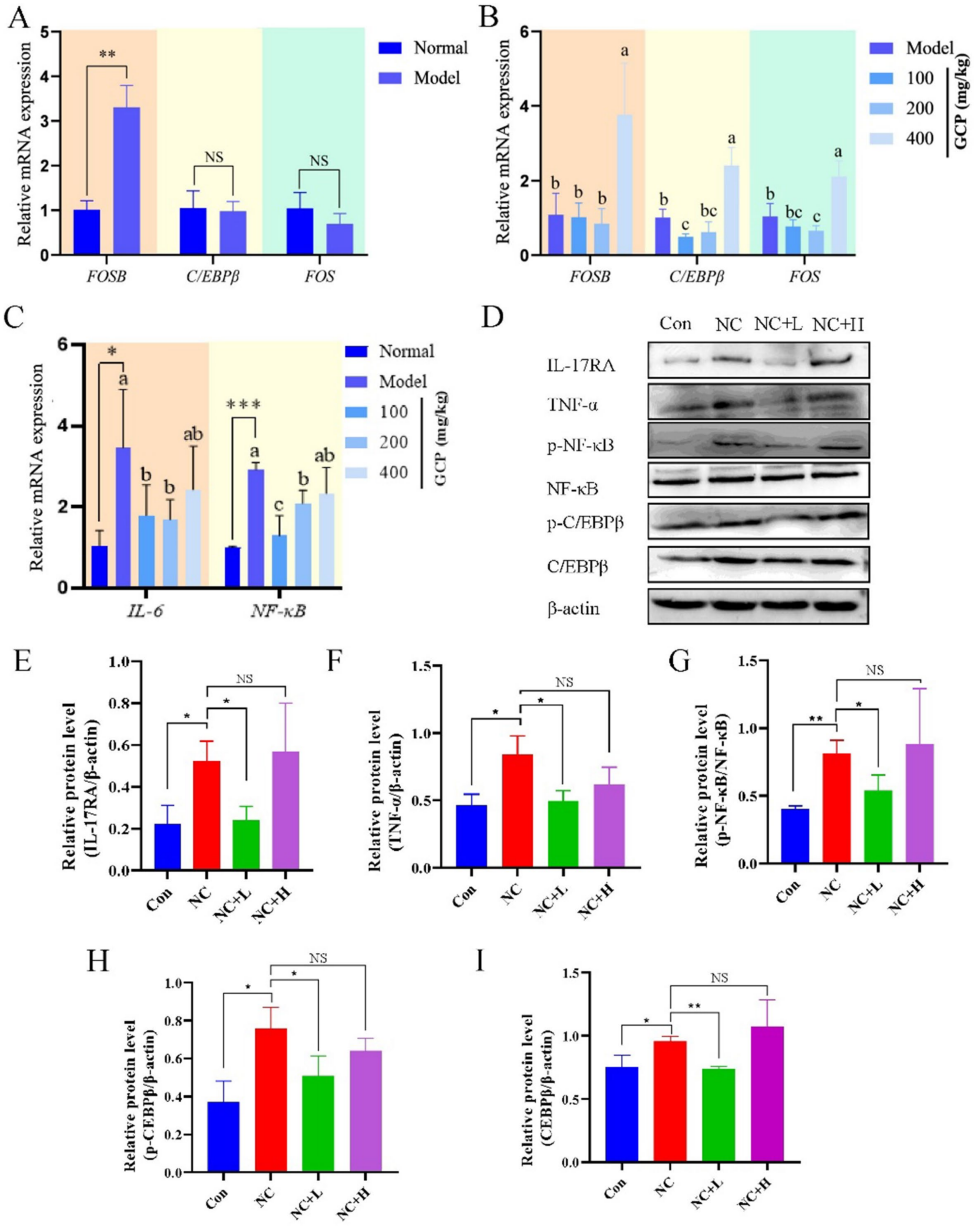
GCP alleviates intestinal injury induced by *NC* through the C/EBPβ-TNF/IL-17 signaling pathway. **(A–C)** Validating *in vitro* transcriptome sequencing data using qRT-PCR. The same letter in the histogram indicates that there is no significant difference between groups (*p* > 0.05), but different letters indicate significant difference (*p* < 0.05). **(D)** Detecting proteins related to the C/EBPβ-TNF/IL-17 signaling pathway using western blot. **(E–I)** Bar graphs show the relative protein levels, with data derived from three independent experiments. Significance: *p < 0.05, **p < 0.01.

To investigate the mechanism by which GCP alleviates *NC*-induced intestinal damage in mice, this study detected relevant biomarkers in duodenal tissues based on transcriptome sequencing. qRT-PCR analysis revealed significantly higher FOSB mRNA levels in the NC group compared with uninfected controls. C/EBPβ and FOS mRNA levels were significantly lower in GCP-treated groups versus the NC group. However, no significant differences in IL-6 or NF-κB mRNA levels were observed between the high-dose GCP group and the NC group (Figures 6A–C). Western blot analysis of proteins associated with the IL-17 and TNF signaling pathways revealed that the expression levels of TNF-*α*, p-NF-κB/NF-κB, IL-17RA, and p-C/EBPβ in the NC group were significantly higher than those in the control group. These elevated protein levels were effectively attenuated by low-dose GCP intervention, whereas high-dose GCP exhibited no significant regulatory effects (Figures 6D–I).

## 4 Discussion

The intestine, a critical organ for digestion, absorption, and immunity, maintains a central role in systemic homeostasis. IECs, the foundation of the intestinal mucosal barrier (18), function not only as barriers but also as frequent primary targets for pathogen attack. For example, *NC*, an obligate intracellular parasite, can penetrate IECs to spread to nucleated cells throughout the host (19, 20). Therefore, understanding the specific mechanisms by which GCP reduces damage to IECs caused by such pathogens holds significant importance for protecting intestinal health.

In this study, BIECs-21 served as an *in vitro* model to investigate cellular responses to *NC* infection. *NC* infection significantly reduced BIECs-21 viability, which was effectively mitigated by GCP pretreatment. LDH release, an indicator of cell membrane integrity, was markedly suppressed by GCP (21), demonstrating its protective effect against *NC*-induced cytolysis.

Transcriptome sequencing was used to screen DEGs in order to examine the interaction mechanisms between *NC* infection and BIECs-21, as well as the mechanism of action of GCP. Transcriptomic profiling and pathway analysis (GO/KEGG) revealed that *NC* infection disrupted immune regulation and signal transduction, particularly activating TNF and IL-17 signaling pathways. *NC* infection elevated transcript levels of IL-17 pathway-associated chemokines (CXCL1/2/3) and inflammatory genes (e.g., FOSB), while GCP pretreatment reduced IL-17/TNF pathway components, including IL-6 and IL1RAP.

TNF-*α*, a critical immune-regulatory cytokine in the TNF signaling pathway, activates the NF-κB and MAPK pathways by binding to TNFR1, thereby mediating cell survival/death signaling and inflammatory responses (22–25). Similarly, IL-17 cytokines (IL-17A-F) enhance antimicrobial defenses and inflammatory reactions by activating the NF-κB, MAPK, and C/EBP pathways (26–28). The AP-1 transcription factor family (e.g., c-Fos, FosB) and C/EBPβ, a member of the C/EBP transcription factor family, bind promoters of inflammatory genes (e.g., IL-6, TNF-α), amplifying inflammatory signals (29, 30). C/EBPβ, a transcription factor common to both TNF and IL-17 pathways, undergoes phosphorylation upon IL-17 stimulation, modulating inflammatory gene expression (31–33).

IL-17A enhances host immune responses to suppress *Trypanosoma cruzi* infection by promoting macrophage microbicidal activity (34, 35). In this study, *NC* infection upregulated FOSB expression, whereas GCP suppressed C/EBPβ and FOS expression, suggesting that GCP alleviates *NC*-induced inflammation by targeting C/EBPβ.

*In vivo*, low-dose GCP attenuated weight loss, mesenteric hemorrhage, and parasite loads in intestinal and cerebral tissues of *NC*-infected mice. Consistent with transcriptome data, *NC* infection elevated duodenal FOSB mRNA and IL-17/TNF pathway-related protein expression, while GCP inhibited C/EBPβ/FOS expression and downstream signaling. Furthermore, GCP significantly reduced both total C/EBPβ protein level and its phosphorylation level, resulting in a decrease in the absolute level of the active phosphorylated form, p-C/EBPβ. These findings indicate that GCP not only inhibits C/EBPβ protein synthesis but also effectively suppresses its phosphorylation. In addition, the findings support the role of GCP in alleviating intestinal damage by modulating C/EBPβ activity. However, high-dose GCP did not demonstrate a therapeutic effect, potentially due to adverse effects on pathways related to gut microbiota and glucose metabolism (36–38).

**Figure 7:**
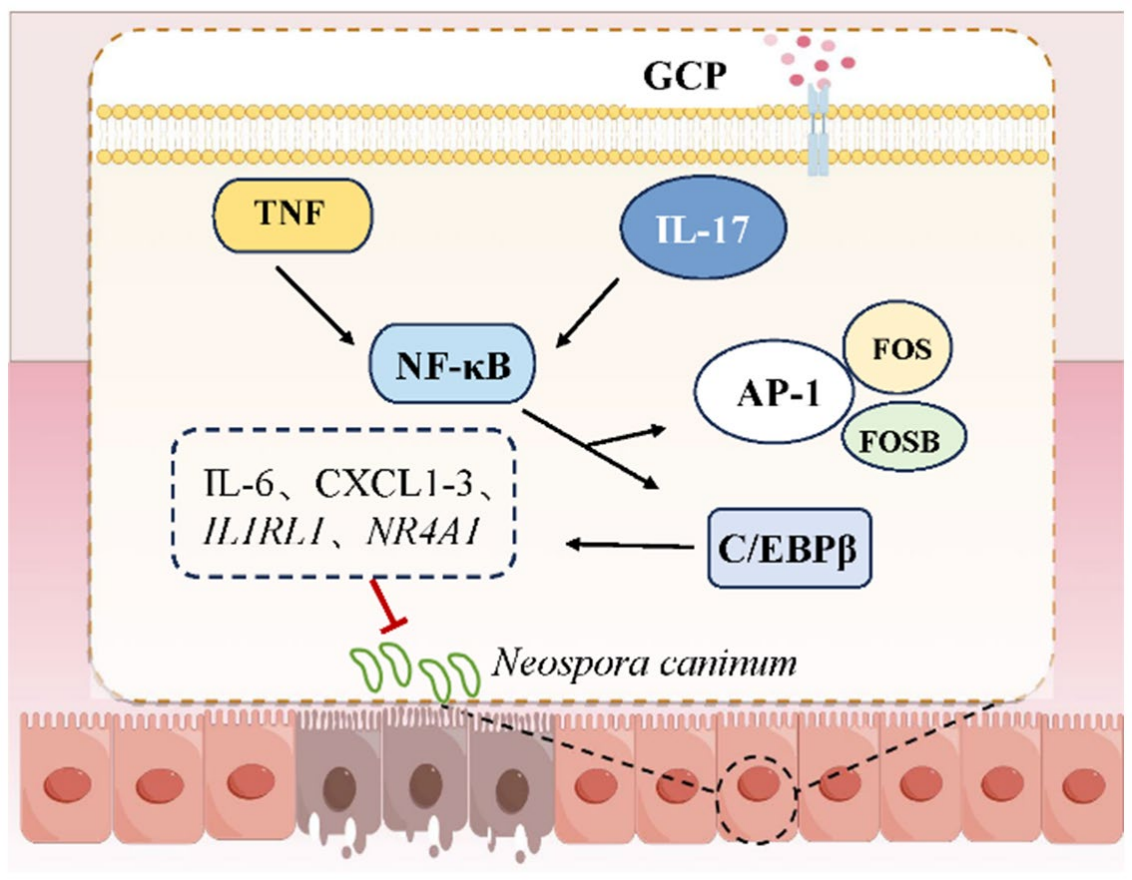
The potential mechanism of GCP relieving *NC* damage to BIECs-21.

Several questions warrant further investigation, including the mechanisms underlying the role of gut microbiota in the anti-*NC* effects of GCP, and the molecular cascades through which GCP regulates the IL-17 and TNF signaling pathways by C/EBPβ. Nevertheless, this study demonstrates that GCP enhances the ability of IECs to resist *NC* infection by modulating immune-related signaling pathways (Figure 7).

## 5 Conclusion

In conclusion, this study provides an *in vitro* model for elucidating the pathogenic mechanisms of host-pathogen interactions and establishes a theoretical foundation for developing natural medicinal agents aimed at preventing and treating pathogen-induced intestinal injury.

## Data Availability

The original contributions presented in the study are publicly available. This data can be found here: https://www.ncbi.nlm.nih.gov/bioproject/PRJNA1398535.
