# Supplementary material for: Glycyrrhiza polysaccharide attenuates Neospora caninum-induced intestinal epithelial cell damage by the C/EBPβ/IL-17/TNF signaling pathway
Source: Front Vet Sci. 2026 Jan 26;12:1753653. doi: 10.3389/fvets.2025.1753653 (PMC12884399; doi:10.3389/fvets.2025.1753653)
Supplement: Supplementary file 1 [file Data_Sheet_1.docx]

**Legends for Supplementary Figures and Table**


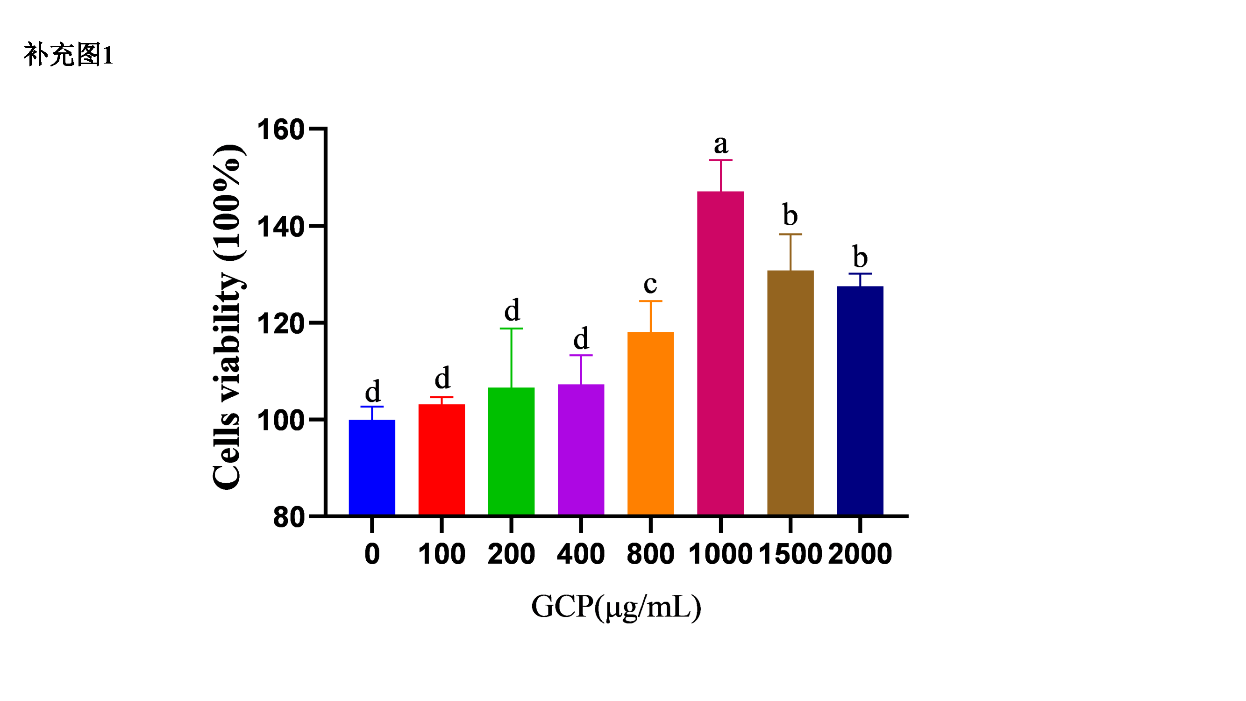


Supplementary Figure 1. Effect of licorice polysaccharide on the viability of BIECs-21 cells.

The same letter in the histogram indicates that there is no significant difference between groups (*p* > 0.05), but different letters indicate significant difference (*p* < 0.05).


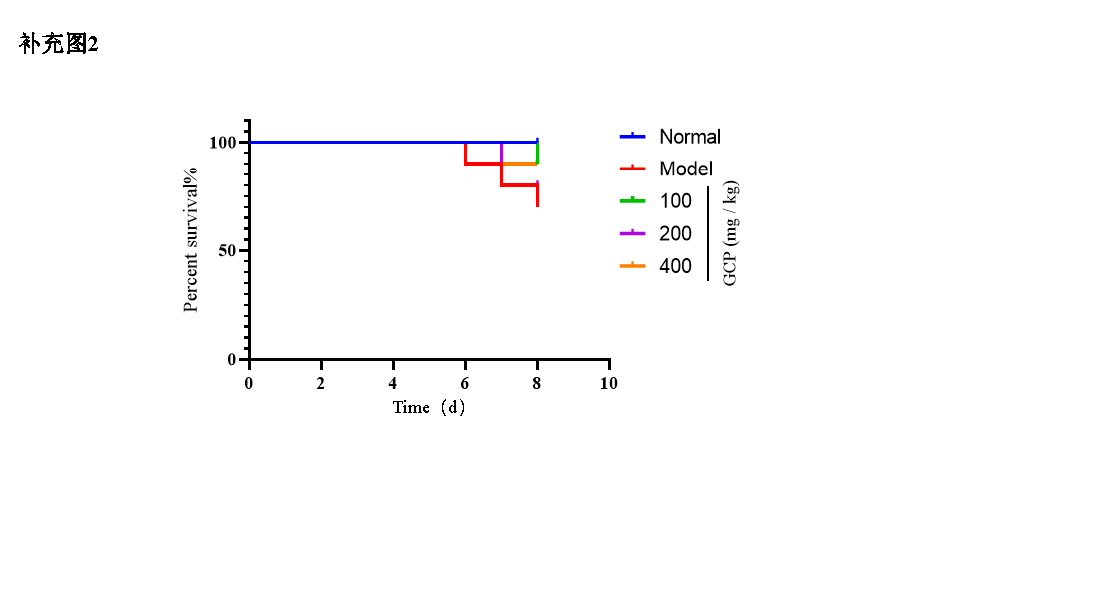


Supplementary Figure 2. Survival rate of mice in each group

Table1. PCR primer sequences for cattle

| Gene | Primer | |  |
| --- | --- | --- | --- |
| *IL1RAP* | | F: 5' CGAGAAAGAACCGGGAGAAGA 3'  R: 5' TCTGCTGAGTGCATCCATTAC 3' | |
| *IL1RL1* | | F: 5' ACATAACGTGCTTGGCTTGC 3'  R: 5' GGATTAAAAGGCAGTCTTTGCGT 3' | |
| *IL18R1* | | F: 5' GGTTCCTGGAGGAGCTGTTGT 3'  R: 5' TCCACTTCAGAACTCTGTGGG 3' | |
| *IL4R* | | F: 5' AAATGACCCCACGGATTTCAG 3'  R: 5' CTCCCAGGTGTCCTCGTAGT 3' | |
| *IL33* | | F: 5' AAAACAAGATCACAAGGATTGCC 3'  R: 5' GCTTTGCCTGCTGAGCATTT 3' | |
| *IL6* | | F: 5' TCCTGAAGCAAAAGATCGCA 3'  R: 5' GGAATGCCCAGGAACTACCA 3' | |
| *ACKR3* | | F: 5' CCGCCAGGCCGTTCG 3'  R: CCTGGATGTTCACCCAGACC 3' | |
| *NR4A1* | | F: 5' GTCTGGATGCAGGAAGCACG 3'  R: 5' TTGGATACAGGGCATCTCAGCTT 3' | |
| *CXCL1* | | F: 5' CGACCAAACCGAAGTCATAGCC 3'  R: 5' TTAAACACAGTCCAGATGGCCC 3' | |
| *CXCL2* | | F: 5' CCAAACCGAAGTCATAGCCA 3'  R: 5' TGGAACAGCCATCCAAGAGC 3' | |
| *CXCL3* | | F: 5' AAACCGAAGTCATAGCCACTC 3'  R: 5' CCTCTATGAGCAGAGACCACT 3' | |
| *FOSB* | | F:5' GGAGAAGAGAAGGGTTCGCC 3'  R: 5' TAGCTGATCTGTCTCCGCCT 3' | |
| *FOS* | | F:5' CGTCAATGCGCAGGACTACT 3'  R: 5' GGAGACTAGGGTGGGCTGTA 3' | |
| *C/EBPβ* | | F:5' TTCCTCTCCGACCTCTTCTC 3'  R: 5' CCAGACTCACGTAGCCGTACT 3' | |
| *β-actin* | | F:5' CCGCAACCAGTTCGCCAT 3'  R: 5' AGGGTCAGGATGCCTCTCTT 3' | |

Table 2 PCR primer sequences for mouse

| Gene | Primer |
| --- | --- |
| *IL-6* | F: 5' GACAAAGCCAGAGTCCTTCAGA 3'  R: 5' TGTGACTCCAGCTTATCTCTTGG 3' |
| *NF-κB* | F: 5' AAGGCCCAGGCGGATATCTA 3'  R: 5' AATCCTGGCTGACTCATGGC3' |
| *FOSB* | F: 5' ATCGACTTCAGGCGGAAACT 3'  R: 5' TTAGCGGATGTTGACCCTGG 3' |
| *FOS* | F: 5' AGAGCGGGAATGGTGAAGAC 3'  R: 5' AGTTGATCTGTCTCCGCTTGG 3' |
| *C/EBPβ* | F: 5' TTATAAACCTCCCGCTCGGC 3'  R: 5' TTCCATGGGTCTAAAGGCGG 3' |
| *β-actin* | F:5' AGCTGAGAGGGAAATCGTGC 3'  R: 5' CTTCTCCAGGGAGGAAGAGGA 3' |
